# A Comparison of Pseudorabies Virus Latency to Other α-Herpesvirinae Subfamily Members

**DOI:** 10.3390/v14071386

**Published:** 2022-06-24

**Authors:** Jing Chen, Gang Li, Chao Wan, Yixuan Li, Lianci Peng, Rendong Fang, Yuanyi Peng, Chao Ye

**Affiliations:** 1 Joint International Research Laboratory of Animal Health and Animal Food Safety, College of Veterinary Medicine, Southwest University, Chongqing 400715, China; cjing1235@163.com (J.C.); li18438695575@163.com (G.L.); w10241229@163.com (C.W.); lyx03014525@163.com (Y.L.); penglianci@swu.edu.cn (L.P.); rdfang@swu.edu.cn (R.F.); 2 Immunology Research Center, Medical Research Institute, Southwest University, Chongqing 402460, China; 3 Chongqing Key Laboratory of Herbivore Science, Chongqing 400715, China

**Keywords:** pseudorabies virus, latency, miRNA, chromatin, immune regulation

## Abstract

Pseudorabies virus (PRV), the causative agent of Aujeszky’s disease, is one of the most important infectious pathogens threatening the global pig industry. Like other members of alphaherpesviruses, PRV establishes a lifelong latent infection and occasionally reactivates from latency after stress stimulus in infected pigs. Latent infected pigs can then serve as the source of recurrent infection, which is one of the difficulties for PRV eradication. Virus latency refers to the retention of viral complete genomes without production of infectious progeny virus; however, following stress stimulus, the virus can be reactivated into lytic infection, which is known as the latency-reactivation cycle. Recently, several research have indicated that alphaherpesvirus latency and reactivation is regulated by a complex interplay between virus, neurons, and the immune system. However, with those limited reports, the relevant advances in PRV latency are lagging behind. Therefore, in this review we focus on the regulatory mechanisms in PRV latency via summarizing the progress of PRV itself and that of other alphaherpesviruses, which will improve our understanding in the underlying mechanism of PRV latency and help design novel therapeutic strategies to control PRV latency.

## 1. Introduction

Pseudorabies virus (PRV) is a member of *Alphaherpesvirinae* under the family *Herpesviridae* that can infect a broad host range of mammals, such as ruminants, carnivores, and rodents. PRV infection mainly causes neurological symptoms and acute death in its non-natural hosts, while causes respiratory disease and reproductive failure in adult pigs and neurological symptoms in piglets, respectively [1]. Recently, it was reported that PRV was isolated from acute encephalitis cases in humans, implying the potential risk of PRV infection from pigs to humans [2]. Although eradication of PRV by the vaccination-DIVA testing strategy has been performed in the United States and several other countries, PRV remains an important pathogen in pig industry in many countries. Particularly, a PRV variant has been reported in China since 2011, which has caused huge economic losses to the Chinese pig industry, making it more difficult for PRV control and eradication [3,4,5].

Alphaherpesviruses, including human herpesvirus (HSV) 1 and 2, varicella zoster virus (VZV), bovine herpesvirus 1 (BHV-1), and pseudorabies virus (PRV), use either lytic or latent infection strategy to infect their hosts. The replication processes and mechanisms involved in their lytic infection have been reasonably well understood [1]. But for latent infection of PRV and other alphaherpesviruses, it is mainly known that the typical feature of virus latency is the existence of viral genome for long periods but no virus progeny production in the infected hosts. After stressful experiences, the virus can be re-engaged in lytic infection, a process known as reactivation [1]. These stressors for PRV reactivation include but not limited to concomitant disease, long-distance transport, poor animal husbandry, and treatment with immunosuppressive agents such as dexamethasone. Those mechanisms involved in PRV latency establishment, latency maintenance, and reactivation from latency remain largely undefined. This review will address features of PRV and other alphaherpesviruses latency with a focus on the potential contribution of viral latency transcripts and viral proteins, viral non-coding RNAs, host immune system, and chromatin epigenetics to virus latency-reactivation cycle.

## 2. The Viral Latency-Associated Transcripts in PRV and Other Alphaherpesviruses Latency

**Figure 1:**
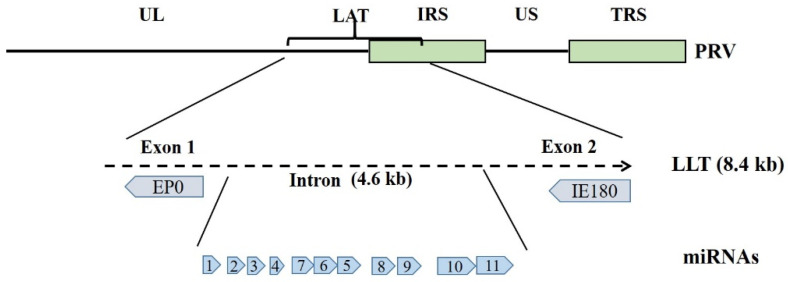
Map of the PRV genome containing unique long (UL), unique short (US), LAT and inverted repeat (IRS and TRS) sequences. The positions of LLT, *EP0*, *IE180*, and miRNAs are annotated, respectively.

During PRV lytic infection, transcription of the viral lytic genes is temporally ordered and known as gene transcription cascade. Viral lytic genes can be subdivided into three classes of successively expressed transcripts, named immediate-early genes, early genes and late genes, respectively [1]. Among them, *IE180* and *EP0* are important regulatory genes during PRV replication. *IE180* gene (homolog of HSV-1 *ICP4*), the only genuine immediate-early gene of PRV, is located in the IRS and TRS repeats and present in two copies in the genome. *IE180* is essential for viral replication in tissue culture, as it is required for the efficient transcription of viral early and possibly late genes. *EP0* is transcribed with early kinetics and functionally homologous to HSV-1 *ICP0*. It is able to activate several viral genes expression such as *IE180*, *UL23,* and *US4*. Although *EP0* is dispensable for viral replication in cell cultures, the viral titers in vitro and virulence in vivo of EP0-defected PRV are significantly reduced [1]. In contrast, when PRV enters a latent infection state, its genome is primarily retained in neurons of the trigeminal ganglion (TG) [6], expression of viral lytic genes is completely inhibited and transcription is restricted to a small region of the viral genome that is named the latency-associated transcript (LAT) locus [7]. The LAT is located at the strand complementary to the *EP0* and *IE180* genes and overlaps the internal repeat sequence of PRV genome [8,9,10]. As reported, various sizes of LATs transcribed from the LAT region can be detected during PRV latency in the infected swine TG [6,11]. The 8.4 kb large latency transcript (LLT) is the largest transcript in the LAT locus, which is then spliced into different sizes to yield a 4.6 kb intron [9,12]. Although the LLT transcript has the potential of encoding proteins (exon 1 and 2), there is no evidence of any protein product in PRV latent infection. In the 4.6 kb region, a cluster of 11 miRNA genes is identified by deep sequencing in porcine dendritic cell and in PK-15 cell line infected by PRV, which suggests a role of the 4.6 kb intron as a primary miRNA precursor [13,14] (Figure 1). Additionally, transcription from the LAT locus can occur either in latent or lytic infection, but a different set of transcripts is expressed in the lytic infection [15].

In HSV-1, its LAT is also expressed during acute and latent infection, and viruses lacking LAT do not establish latency as efficiently as wild-type HSV-1 and exhibit delayed reactivation [16,17,18], suggesting that the LAT is involved in HSV-1 latency establishment and facilitates HSV-1 reactivation from latency. In VZV, although a latency-associated transcript that lies antisense to the viral transactivator gene 61 (*ORF61*) predominates during virus latency [19], transcripts from 12 VZV genes (e.g., *ORF63*) are identified in human ganglia removed at autopsy. But it is difficult to ascribe these as transcripts present during latency as virus reactivation may occur in the post-mortem time period in the ganglia [20]. Although the transcription of several LATs and relevant genes in alphaherpesviruses latently infected neurons is evident, their functions in virus latency and reactivation are still not clear.

## 3. Viral Non-Coding RNAs in PRV and Other Alphaherpesviruses Latency

Most herpesviruses produce various non-coding RNAs such as LAT-encoded miRNAs during virus latency. Several HSV-1 miRNAs have been found to reduce the expression of viral lytic regulatory proteins ICP0 and ICP4, suggesting that the viral miRNAs may facilitate establishment and maintenance of viral latency by post-transcriptional regulation of viral lytic gene expression [21]. As for PRV, previous studies found that the intron region of LLT functioned as a primary miRNA precursor, which encodes a cluster of 11 miRNA genes [13,14]. To assess the importance of this miRNA cluster in establishment of PRV latency in swine, Mahjoub et al. generated a PRV mutant with a 2.5-kb portion deletion in the LLT intron harboring 9 miRNA genes. This mutant displayed almost identical acute infection properties to those of parental PRV in vitro and in vivo. It also successfully establishes latency in vivo, demonstrating that these miRNAs are nonessential for PRV infection and latency establishment in TG. However, massive host gene upregulation is found in TG during miRNA-deleted PRV latency, and several biofunctions including those related to cellular immune response and dendritic cells migration are impaired during this process [12]. These findings supported a function of PRV LAT-encoded miRNAs in the modulation of host response for maintaining a latent state. However, the role of these miRNAs in PRV reactivation from latency has not been explored, hence further studies are needed to determine whether these miRNAs play a role in the process of PRV reactivation.

Similarly, initial study on HSV-1 has found that LAT-encoded miRNAs can be detected in latently infected TGs, and the LAT promoter mutants lacking miRNA expression can still replicate normally and establish wild-type levels of latency, suggesting that these viral miRNAs might be dispensable for HSV latency and reactivation [22]. However, further study focusing on the individual miRNA function has found that single miRNAs might play important roles in HSV-1 latent infection. The miR-H2, one HSV-1 LAT-encoded miRNA molecule, is found antisense to the *ICP0* gene and appears to reduce ICP0 expression [21]. To further examine the importance of miR-H2 in HSV-1 latency-reactivation cycle, researchers used a codon redundancy strategy to construct a miR-H2 disrupted mutant without any changes in the overlapping *ICP0* gene [23]. Compared to its parental virus, ICP0 protein was much more expressed during infection, confirming that miR-H2 downregulated HSV-1 *ICP0* gene expression. Ocular infection in mice showed that miR-H2 mutant increased neurovirulence as judged by mouse survival experiment. Intriguingly, this miR-H2 KO mutant is reactivated significantly earlier than its parental strain in the mouse explant TG reactivation model, indicating that the miR-H2 is involved in reducing HSV-1 neuroinvasion and maintaining HSV-1 latency [23,24].

Besides encoding multiple miRNAs in LAT, two small noncoding RNAs (sncRNAs) are also encoded in HSV-1 LAT region. The two sncRNAs (sncRNA1 and sncRNA2) are 62 and 36 bp in length, respectively, presenting antiapoptotic activity in vitro [25,26]. Furthermore, expression of these sncRNAs by transient transfection assays inhibits cell apoptosis and virus productive infection in vitro [27]. Additionally, these two sncRNAs were able to induce beta interferon promoter activity and cell survival in vitro [28]. All of these results suggest that sncRNAs play important roles in HSV-1 productive infection in vitro, while its role in virus latency and pathogenesis in vivo is not clear. Recently, researchers constructed a recombinant HSV-1 lacking the 62-bp sncRNA1 region in wild-type strain McKrae using dLAT2903 (LAT-minus) virus and compared this mutant virus with its parental virus and McKrae in vitro and in vivo. The replication kinetics of sncRNA1 mutant virus were similar to that of dLAT2903 and McKrae. Meanwhile, ocular virus titers, eye disease, and levels of latency and reactivation were similar in mice infected with either McKrae or the mutant virus. However, absence of sncRNA1 significantly decreased the ocularly infected mice survival and led to virus increased virulence [29]. These suggest that sncRNA1 has a protective function during acute ocular infection, although the presence of sncRNA1 sequence has no significant effect on virus latency or reactivation. Yet it remains to be determined whether PRV encodes the similar sncRNAs in LAT region and how these potential sncRNAs function in virus latency.

## 4. The Viral Proteins Directly and Indirectly Participated in Alphaherpesviruses Latency

Several viral proteins can play important roles in alphaherpesviruses neuroinvasion. For years, studies upon these proteins have highlighted their potential functions in virus latency and reactivation process directly and indirectly (Table 1). For example, thymidine kinase (*TK*) is an important virulence gene of alphaherpesviruses. The relationship between TK activity and HSV-1 virulence has been characterized in a mouse model. Following corneal inoculation, the *TK* (+) strain produced high central nervous system (CNS) titers and established latency in 78% of surviving mice. In contrast, the *TK* (−) strain did not invade the CNS or establish latency [30]. In another study, the HSV-1 *TK* (−) mutant (F)A305 showed reduced neurovirulence and failed to establish latency in mice, but was able to establish latency in rabbits [31]. Contrary to the above studies, Coen et al. found that *TK* (−) mutants established latent infections in mouse TG, but were severely impaired for acute replication in TG and failed to reactivate from ganglia upon cocultivation with permissive cells [32]. It is indicated that *TK* appears to be necessary for HSV-1 reactivation and unnecessary for latency establishment.

To determine the ability of HSV-2 *TK* (−) strain to establish latency and reactivate in vivo, an intranasal or ocular infection model in rabbits was used [33]. The results showed that the *TK* (−) strain can replicate and shed virus in the eye after intranasal or ocular infection, and can cause acute keratitis and establish TG latency. Moreover, 30% of rabbits initially infected in the eyes reactivated following drug-induced immunosuppression with latently infected control rabbits uniformly reactivated. The study shows that the *TK* gene is important but not essential for latency or reactivation in this model [33]. In the relevant studies of BHV-1, a *TK* (−) strain was initially found to be incapable of establishing latency in rabbits following intranasal infection [34]. However, later it was demonstrated that after intranasal infection, latent infection established by a BHV-1 *TK* (−) strain was reactivated by dexamethasone [35]. Similarly, an experiment was conducted to examine the neuroinvasiveness of PRV *TK* (−) mutant in pigs. Results showed that mutations in *TK* gene were associated with reduced virus virulence and replication in peripheral target tissues, and also reduced migration to the olfactory and trigeminal pathways [36]. In addition, *TK* defective vaccine strain of PRV established latency in swine TG, however, dexamethasone treatment failed to reactivate the latent *TK*-defected PRV in all ten pigs. In contrast, the PRV *TK* (+) strain can be reactivated from 54 of 55 latently infected pigs following dexamethasone treatments [37]. All above suggests the effectiveness of the dexamethasone treatments experiment for PRV reactivation, and the crucial role of *TK* gene in the latent PRV reactivation process.

Homologous proteins of PRV gE are found in all members of the *alphaherpesvirinae*, suggesting a conserved biological function of this protein. Studies have revealed that homologous proteins of gE among alphaherpesviruses including PRV, HSV-1, and BHV-1 are not required for replication of these viruses in cell culture. In addition, the gE proteins of PRV and HSV-1 can induce cell fusion and promote the spread of virus from cell-to-cell [38]. VZV lacking *gE* also shows significant restriction in cell-to-cell spread and reduced yield of infectious virus production [39]. More importantly, gE is a critical virulence determinant of the alphaherpesviruses. Deletion of *gE* from HSV-1, PRV, and BHV-1 drastically decreases HSV-1 neurovirulence in mice, results in PRV virulence and spread reduction in the nervous system and abolishes BHV-1 virulence in calves, respectively [38]. These findings provide strong evidence that the gE protein plays important roles in cell-to-cell spread, invasiveness, and virulence of alphaherpesviruses. Moreover, gE plays a role in alphaherpesviruses (e.g., PRV, HSV-1, and BHV-1) latency. The *gE*-deleted strain of these viruses can stay latency in TG, while the ability to replicate in all levels of host neurons were significantly reduced, so *gE*-deleted mutants establish latency in neurons less efficiently, and therefore are less likely to be reactivated than wild-type virus [38]. Further studies upon HSV-1 and PRV demonstrated that membrane proteins gE, gI, and US9 promote their anterograde transport. Anterograde transport is a process that the reactivated virus particles return to epithelial tissues by fast axonal transport moving from the neuron cell body to axon termini, which plays a key role in HSV-1 and PRV reactivation from latency. Virus transport in the anterograde direction contains at least two processes: (1) The anterograde transport in axons from cell body to axon termini, (2) virus egress from axon termini and subsequent virus spread into epithelial cell [40]. It has been shown that all *gE*-, *gI*-, and *US9*- mutants of HSV and PRV have substantial defects in anterograde transport. However, gE/gI formed protein complexes and exerted major effects in spread of virus from axon termini to epithelial cell, whereas US9 only participated in axonal events due to its lack of extracellular domain. gE/gI and US9 in both HSV and PRV act primarily in the cytoplasm to promote virus assembly and virus particles sorting into axons, which also supports the observations that HSV *gE*-/*US9*- and PRV *US9*- mutants are transported normally in axons but defected in anterograde transport [40].

In addition, the *EP0* gene of PRV is a homolog of HSV-1 *ICP0*, which has been reported to be involved in the reactivation of HSV-1. Upon deletion of *EP0* in PRV genome, the latent viral DNA level in swine TG is reduced and subsequently the reactivation of virus is also inhibited [1,41]. In a similar study, an *EP0*-deficient mutant was able to establish latency in mouse TG, but latent virus cannot be reactivated in explant reactivation assays [42]. Inactivation of the *EP0* gene inevitably results in a mutation of LLT, which is located on the complementary DNA strand of *EP0*. Therefore, it is uncertain whether the *EP0* or LLT mutation might play a role in regulating the latency and reactivation state, but at least it suggests that the *EP0* gene locus is crucial for PRV latency and reactivation in TG. The IE180 protein is a major regulator of global gene expression of PRV. Research has found that during PRV reactivation, IE180 is readily expressed and might play important roles in PRV reactivation [43]. Furthermore, IE180 forms protein–DNA complex at the transcription start site of LAT, and therefore suppresses the activity of PRV LAT promoter by interacting with a TATA box within the promoter, which might be important for swiching from latency to reactivation state [44].

In addition, a 2.0-kb RNA transcribed from PRV LLT was found to carry polyadenylation signal (AATAAA) during virus lytic infection and therefore could be translated. The RNA contains two potential exons capable of encoding a 20-kDa ORF1 and a 47-kDa ORF2. The putative ORF1 protein presents 44% homology to the apoptosis repressor ARC, 36% homology to a protein kinase C substrate, and 30% homology to a serine/threonine protein kinase. In contrast, the predicted ORF2 protein has no homology among any known proteins [15]. It would be interesting to determine if either putative protein is actually expressed, and plays crucial roles during a productive or latent infection. Unfortunately, in recent years no further advances have been made on the roles of LLT-encoded proteins in PRV latency. However, a series of relevant progress have been obtained in BHV-1 (Table 1). Like other alphaherpesviruses, BHV-1 latency related (LR) gene, a gene similar to PRV LAT, is abundantly expressed in latently infected neurons [45]. Two potential open reading frames (ORF 1 and 2) are located in the BHV-1 LR gene [46]. In exploring the role of ORF1 and ORF2 in virus latency, researchers have constructed a BHV-1 mutant with three stop codons at the N-terminus of ORF2, which resulted in ORF2 expression abolishment and ORF1 expression reduction [47,48]. This mutant failed to reactivate from latency following dexamethasone treatment [49], suggesting that protein expression within the LR gene is important for virus latency and reactivation. Moreover, BHV-1 ORF2 can participate in multiple cellular processes to regulate virus latency-reactivation cycle. For example, abolishment of ORF2 expression in the above BHV-1 mutant induces higher levels of apoptosis in infected neurons, which is considered an important cellular process in regulating the latency-reactivation cycle [50,51]. In addition, Notch family members are membrane-tethered transcription factors that regulate neuronal maintenance, development, differentiation, and development of nearly all non-neuronal cells [52]. Notch1 activates the BHV-1 immediate early transcription unit 1 and *ICP0* early promoters as a cellular transcription factor and enhances BHV-1 productive infection. ORF2 is able to interact with the components (Notch 1 and 3) of the Notch signaling pathway in a yeast two-hybrid screen, and therefore consistently interferes with Notch1–3-mediated transactivation of cellular promoters [53,54]. Furthermore, distinct domains in ORF2 are crucial for interfering with Notch1-mediated transactivation of the *ICP0* early and glycoprotein C promoters. Given the important regulatory role of ICP0 (EP0) in both virus latent and lytic infection cycles, the interference effect of ORF2 might inhibit virus productive infection and promote latency establishment [55]. So far, however, no proteins similar as ORF1 and ORF2 in BHV-1 have been identified in PRV LAT. Hence, future studies could focus on roles of PRV LAT-associated proteins in the regulation of PRV latency-reactivation cycle.

## 5. Immune Regulation of PRV and Other Alphaherpesviruses Latency

The latency-reactivation cycle of alphaherpesviruses is considered to be tightly regulated by a subtle and an incompletely understood interplay between the virus, the neuron, and the immune system [56]. Moreover, it is becoming increasingly evident that both innate and adaptive immunity play important roles in the initial control of herpesviruses infection and latency establishment [57]. In host innate immunity, type I interferons (IFNs) containing IFN-α and -β-mediated immune responses serve as the front line of host defense against virus infection. Activation of the type I IFN pathway is transduced via Janus kinases (JAKs) and may result in the initiation of inflammatory response and expression of antiviral genes such as IFN-stimulated genes (ISGs). IFNs have been reported to play important roles in limiting alphaherpesviruses replication and spread [58,59,60]. Furthermore, IFN-α has been demonstrated to drive both HSV-1 and PRV into a latency-like quiescent state in in vitro cultures of porcine TG neurons [61]. IFN-α treatment results in suppression of the immediate-early protein ICP4 of HSV-1 or its counterpart IE180 in PRV, which might be a key step for type I IFNs in promoting the establishment of alphaherpesviruses latency [61]. In addition, a recent investigation highlighted the regulatory role of type I IFNs signaling in the relationship between HSV-1 genomes and the nuclear environment during latency establishment. It showed that without IFN-α treatment, HSV-1 infection led to the formation of replication compartment genome pattern (the lytic infection pattern) in the nucleus of ~91% neurons. However, IFN-α treatment favored the multiple latency-like pattern (the latent infection pattern), with 82% neurons showing this pattern. Moreover, nearly all HSV-1-infected TG neurons harvested from type I IFN receptor KO mice showed the formation of replication compartment pattern irrespective of treatment with IFN-α. Hence, the type I IFNs favors the formation of latency-like genome pattern in HSV-1-infected neurons [62]. Nevertheless, HSV-1 has evolved strategies to inhibit type I IFNs signaling. Its LAT transcript was shown to delay IFN-α and -β expression during acute infection. Further studies found that JAK-1 and -2, as well as several downstream effectors of the JAK pathway, were also downregulated in a LAT-dependent manner during HSV-1 latency [63]. Recently, studies indicated that PRV has developed multiple strategies to antagonize the type I IFNs-mediated innate immunity via several viral proteins, ranging from inhibiting pattern-recognition receptors induced type I IFNs production to antagonizing the IFN signaling pathway and neutralizing the antiviral functions of ISGs [64,65,66,67,68]. This feature might confer PRV the ability of reactivation from latency in neurons.

Peripheral neurons are the main sites for alphaherpesviruses latency, virus latency involves a precise interaction between the virus and neuron immunity. Research shows that sympathetic neurons can be cultured as a pure population with the treatment of nerve growth factor (NGF). Meanwhile, latency can be established in primary sympathetic neurons cultured in presence of NGF. Furthermore, the phosphatidylinositol 3-kinase (PI3-K) pathway triggered by NGF-binding to the TrkA receptor tyrosine kinase (RTK) is crucial in maintaining HSV-1 latency by using a primary neuronal culture system. The PI3-K p110α catalytic subunit is specifically required to activate 3-phosphoinositide-dependent protein kinase-1 (PDK1) and maintain virus latency. Depletion of PDK1 results in HSV-1 reactivation. Thus, the RTK/PI3-K/PDK1-signaling is a critical host element that regulates the HSV-1 latent-lytic switch [69]. In addition, PRV also establishes a reactivatable, quiescent infection in neurons cultured in modified Campenot tri-chambers. Further study shows that several host factors including protein kinase A (PKA) and c-Jun N-terminal kinase (JNK) in cell bodies prevent establishment of quiescent infection and promote productive replication of axonally delivered virus genomes. Treatment with forskolin (a potent adenylate cyclase activator) on cell bodies activates both PKA and JNK and therefore results in virus productive infection. However, virus lytic infection can completely lost when PKA and JNK activities are inhibited [70]. Therefore, PKA and JNK signals significantly affect the switch of PRV latent and lytic infection.

In host adaptive immunity, the γδ T cells play a protective role in the immunosurveillance against alphaherpesviruses infection. Study upon HSV-1 infection in TCRγδ- or TCRαβ-deficient mice have shown that γδ T cells limit HSV-1-induced epithelial lesions and protect mice from HSV-induced lethal encephalitis, which is resulted from the γδ T cell-mediated arrest of virus replication and neurovirulence [71]. Furthermore, γδ T cells and macrophages are able to infiltrate into the TG in HSV-infected mice, and then several cytokines in inhibiting HSV-1 replication, for example IFN-γ, TNF-α and IL-12, are expressed, suggesting a direct role of γδ T cells in controlling virus replication [72]. Moreover, several studies indicate that CD4^+^ and CD8^+^ T cells might play crucial roles in restricting HSV-1 infection [73,74], and a study using two different pathogenic strains of HSV-1 showed that the high pathogenic strain induced a stronger CD4^+^ and CD8^+^ T-cell response in the draining lymph nodes and latently infected TG. It was proposed that greater viral gene expression by the high pathogenic strain during latency might result in a larger T-cell infiltrate in both the cornea and the TG [75]. Further studies found that CD8^+^ T cells inhibited HSV-1 replication in peripheral ganglia and prevented HSV-1 reactivation without destroying the infected neurons [76,77]. It was also reported that CD8^+^ T cells might prevent HSV-1 reactivation from latency at least in part through the secretion of IFN-γ [57]. First, IFN-γ mRNA and protein were consistently detected in latently infected ganglia [78]. Subsequently, treatment with IFN-γ could block HSV-1 reactivation from latency on cultures of latently infected TG in the early reactivation process [79]. Lastly, although knockout of IFN-γ and IFN-γR from mice failed to affect HSV-1 replication and latency establishment in TG, the incidence of stress-induced reactivation in mutant mice was significantly higher than in control mice, suggesting the crucial role of IFN-γ in inhibiting HSV-1 reactivation from latency [80].

As described above, ICP0 is a crucial transactivator that is required for efficient HSV-1 reactivation from latency. The cyclin-dependent kinase (cdk) is required for the post-translational modifications necessary for ICP0 transactivating activity [81]. Study has indicated that IFN-γ could induce production of the cdk inhibitors, which might block HSV-1 reactivation from latency probably by inhibiting ICP0 transactivating activity [81,82]. Additionally, expression of viral proteins is not completely silent but sporadically expressed at low levels during HSV-1 latency, which can be then recognized by ganglion-resident HSV-1-specific CD8^+^ T cells. In a recent study, several HSV recombinants that have different viral promoters driving expression of the immunodominant gB epitope (gB_498–505_) were constructed. They induced equivalent ganglionic CD8^+^ T-cell responses but contained altered gB-CD8s (CD8^+^ T cells that recognize gB_498–505_) immunodominance during latency. The results indicate that the selection of epitope promoter could influence CD8^+^ T-cell population hierarchies and their function. Since CD8^+^ T cells can influence lytic/latent cycles in reactivating neurons, it is convinced that improving their ganglionic retention and function may offer a strategy in vaccine design to reduce virus reactivation [83]. In addition, CD8^+^ T cells can be exhausted during viral chronic infection, which is characterized by reduced memory potential, sustained expression of inhibitory receptors, and poor effector function [84,85]. Moreover, successful reactivation of the latently infected HSV-1 from TG was assumed to be partly dependent on the exhausting CD8^+^ T cells [86,87]. Furthermore, it was found that HSV-specific CD8^+^ T cells from symptomatic patients were phenotypically and functionally exhausted, and the exhaustion receptors (PD-1, LAG-3, TIGIT, and TIM-3) were up-regulated on HSV-specific CD8^+^ T cells. Moreover, blocking of LAG-3 and PD-1 synergistically restored anti-viral CD8^+^ T-cell responses, reduced HSV-1 reactivation from latency, and prevented UV-B irradiation induced recurrent ocular herpetic infection and disease in latently infected mice [88].

The role of CD4^+^ T cells in regulating HSV-1 latency is another area that needs to be explored. One recent study demonstrated that lack of CD4^+^ T cells during the initial programming of HSV-specific CD8^+^ T cells caused transient partial exhaustion of CD8^+^ T cells characterized by elevated PD-1 levels and hence reduced the ability to control HSV-1 latency in sensory ganglia. This finding demonstrates that CD4^+^ T cell is important for generation of a memory CD8^+^ T-cell population, which provides immune surveillance for HSV-1 latency [89]. A recent study also found that HSV-1 infection triggered the activation of regulatory T cells (Treg cells). In an HSV-1 ocular infection model, researchers observed a strong correlation between the level of Treg cells and virus infectivity [90]. Furthermore, depletion of Treg cells largely limited HSV-1 latency establishment. Stress-induced HSV-1 reactivation was tightly dependent on enhanced Treg cell functions, which can suppress the immune surveillance by CD8^+^ T cells and permit viral replication. Taken together, Treg cell may serve as a key target for controlling HSV latency and reactivation [90].

As for PRV, the relevant study upon immune modulation of its latency-reactivation cycle is obviously lagged behind and mainly accompanied by PRV vaccine research. As is known, modified live vaccines have been successfully used to control PRV infection for many years [91]. However, vaccination with various live attenuated strains did not induce complete inhibition of a latent PRV infection; meanwhile, live vaccine strains are able to establish latency themselves [91,92]. Furthermore, the target neurons cannot be superinfected with the wild-type virus once latently infected by a preceding live vaccine strain [93]. In addition, although the significance of CD4^+^ T cells in protective immunity against PRV infection was demonstrated in a murine model [94], the potential role of T cell and its secreted cytokines in PRV latency-reactivation cycle still needs to be determined. Taken together, it is suggested that the immune response induced by modified live vaccines might play important roles in controlling wild-type PRV latency.

## 6. Chromatin Regulation of Alphaherpesviruses Latency

**Figure 2:**
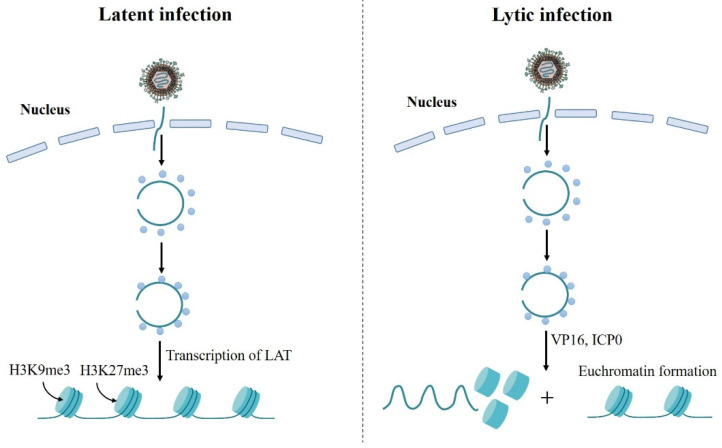
Chromatin can play important roles in regulation of alphaherpesvirus latent and lytic infection. During latent infection of neuronal cells, the virus capsid is transported to the nuclear pore where viral genome is released into the nucleus. When the genome enters the nucleus, it can be rapidly circularized and complexed with histones. Then the LAT is expressed and it promotes the association of heterochromatin marks on the viral genome (**left panel**). During lytic infection of epithelial cells, the virus capsid is also transported to the nuclear pore where the viral genome is released into the nucleus. In the nucleus, viral genome rapidly circularizes and becomes associated with histones. Then VP16 can decrease histone association with viral IE genes and increase euchromatin marks on the remaining histones. ICP0 can be expressed and it promotes the same processes as VP16 on the viral genome (**right panel**).

Chromatin epigenetics, the regulation of nucleosomes and chromatin structure on the genome, is an important determinant of cellular transcription, replication, and differentiation. Recently, several studies have found that chromatin plays an important role in the regulation of alphaherpesviruses latent and lytic infection [95] (Figure 2). During HSV-1 latency, the viral DNA persists as an episome assembled in nucleosomal chromatin structure in sensory neurons of peripheral ganglia [96,97]. Additionally, chromatin immunoprecipitation (CHIP) assay shows that HSV lytic gene promoters are complexed with modified histones associated with heterochromatin during latency establishment, and transcription of the LATs promotes the formation of heterochromatin on viral lytic gene promoters [98,99]. Furthermore, the viral genome is enriched for several chromatin modifications, including the histone H3 lysine 9 trimethyl (H3K9me3), H3K9 dimethyl (H3K9me2), and H3 lysine 27 trimethyl (H3K27me3) modifications [98,99,100], and H3K27me3 is a major form of heterochromatin on the viral genome [99] (Figure 2). During lytic infection, there are no histones present in the virion before infection [101,102]. Once the viral DNA enters the cell nucleus, host cell could assemble chromatin on the naked viral DNA to silence incoming genes, as is observed for transfected DNA [103]. However, there are few nucleosomes on viral genome during virus replication period as demonstrated by nuclease-digestion assays. It is found that viral DNA replicates and accumulates in replication compartments that involves disruption of the host chromatin [104,105]. Moreover, several viral proteins are involved in reducing viral DNA chromatin. VP16, the virion transactivator protein, has been found to recruit the chromatin-modifying co-activators and underrepresentation of histones at immediate-early gene promoters in addition to recruiting transcription factors to immediate-early gene promoters [106]. *VP16* defective mutant also showed increased heterochromatin association with viral lytic promoters [106]. Therefore, VP16 plays an important role in reducing total chromatin levels on immediate-early genes during lytic infection. Meanwhile, ICP0 expression during HSV lytic infection results in an increase in euchromatin formation at the lytic genes and a decrease in total histone in association with immediate-early and early gene promoters [107]. *ICP0* defective mutant shows increased heterochromatin association with viral lytic promoters [108], indicating that ICP0 contributes to both histone removal at HSV lytic genes and modifications on histones assembled on viral DNA during lytic infection.

For that of PRV, Zhang et al. establishes a detection method combined CHIP with qPCR to determine the chromatin status of PRV in host cell during lytic infection. The results show that PRV genome is associated with histone H3 and several segments of the genome are presented as chromation state [109]. In addition, IE180 is able to potentiate the activity of the major late promoter in a reconstituted chromatin assembly system. Function of IE180 requires the simultaneous action of transcription factor IID (TFIID) and results in the formation of stable preinitiation complexes within nucleosome-assembled DNA. Meanwhile, in order to stimulate subsequent transcription, IE180 is required from the onset of nucleosome assembly and it is uncapable of reversing nucleosome-mediated repression once this repression established [110]. These results indicate that IE180 stimulates TFIID binding to promoters and competes with nucleosomes during chromatin reconstitution. However, the potential regulation mechanisms of chromatin in PRV latency are still unclear, which need to be further explored in the future.

## 7. Conclusions and Prospects

Modified live vaccines against PRV have been successfully used for many years in pig industry. Consistent with this, PRV in pigs has been well controlled and eradicated by the use of vaccination in the United States and many European countries. However, the genetically different PRV variants have emerged in China since late 2011, which makes PRV control more difficult [3,4,5]. Additionally, PRV establishes a lifelong latent infection in peripheral nervous system in pigs, which is similar as other alphaherpesviruses. Vaccination with various live attenuated strains always fail to induce complete inhibition of a latent PRV infection. After stress stimulus, the latently infected PRV is able to reactivate, shed, and spread in susceptible pigs. Therefore, understanding the mechanisms behind PRV latency-reactivation cycle is important for the control and eradication of PRV infection.

Nevertheless, the relevant advances in PRV latency are largely lagged behind compared to other alphaherpesviruses such as HSV-1. An important reason is that PRV latency cannot be stably established in the mouse model due to the severe lethality (~100%) of PRV in mice. Therefore, studies upon PRV latency in vivo have to be conducted in pigs, which make the relevant study more expensive and unmanageable. Hence, to establish more convenient and stable animal model for PRV latency research, specific gene-edited transgenic mice suitable for PRV latency and reactivation might be an important direction to promote the related research in future. Although most studies on PRV latency and reactivation involve animal models, in vitro models have been developed to provide a more convenient approach for understanding the molecular biology of PRV latency. Most of the in vitro models use dissociated neuron cultures to establish a quiescent infection, and the isolated neurons usually need to be pretreated with IFN or replication inhibitors to block the initial productive infection. Recently, Koyuncu et al. used a modified Campenot tri-chambers to physically separate axons from their cell bodies. By culturing primary neurons derived from the superior cervical ganglia of rat embryos in these chambers, they successively established reactivatable quiescent infections by PRV without treatment of any replication inhibitor or cytokine [70,111]. This technique provides an important reference tool for the relevant study of PRV latent infection in vitro.

In addition, referring to the related research progress of HSV-1 and other alphaherpesviruses, researchers can explore the mechanisms of PRV latency and reactivation from aspects of PRV-encoded regulatory miRNA, LAT-encoded unknown regulatory proteins (e.g., the analogues of BHV-1 ORF2), other viral proteins involved in virus latency or reactivation, both innate and adaptive host immune regulation and chromatin epigenetics on viral genome. It is convinced that the continuous discovery of PRV latency mechanisms will contribute to proposing novel therapeutic methods for PRV control in future.

## Figures and Tables

**Table 1:** The viral proteins participated in alphaherpesviruses latency as described in this study.

Viral Genes	Viral Proteins	Virus	Proposed Function
*UL23*	Thymidine kinase (TK)	HSV-1	Necessary for HSV-1 reactivation and unnecessary for latency establishment
		HSV-2	Important but nonessential for latency or reactivation
		BHV-1	Probably unnecessary for latency-reactivation cycle
		PRV	Crucial for the latent PRV reactivation
*US8*	gE	HSV-1/PRV/BHV-1	Important for efficient virus latency establishment and reactivation in neurons due to its capacity for promoting virus neuroinvasion
*US9*	11K	HSV-1/PRV	Affect virus reactivation due to its critical role in promoting virus anterograde transport in neurons
*EP0*	EP0	HSV-1/PRV	Crucial for the latency and reactivation
*IE180*	IE180	PRV	Has important roles in PRV reactivation; Important for swiching from latency to reactivation state
*ORF1*	ORF1	BHV-1	Might be important for the virus latency and reactivation
*ORF2*	ORF2	BHV-1	Inhibits apoptosis; interferes with Notch1-mediated transactivation of ICP0; probably inhibit virus productive infection and promote virus latency

## Data Availability

Not applicable.
